# Intracellular *Streptococcus pneumoniae* develops enhanced fluoroquinolone persistence during influenza A coinfection

**DOI:** 10.3389/fmicb.2024.1423995

**Published:** 2024-07-05

**Authors:** Mirelys Hernandez-Morfa, Nicolas M. Reinoso-Vizcaino, Victoria E. Zappia, Nadia B. Olivero, Paulo R. Cortes, Cinthia C. Stempin, Daniel R. Perez, Jose Echenique

**Affiliations:** ^1^Centro de Investigaciones en Bioquímica Clínica e Inmunología (CIBICI)-Consejo Nacional de Investigaciones Científicas y Técnicas (CONICET), Córdoba, Argentina; ^2^Departamento de Bioquímica Clínica, Facultad de Ciencias Químicas, Universidad Nacional de Córdoba, Córdoba, Argentina; ^3^Department of Population Health, College of Veterinary Medicine, University of Georgia, Athens, GA, United States

**Keywords:** *Streptococcus pneumoniae*, influenza A, oxidative stress, stress response, intracellular survival, persistence, fluoroquinolones, autophagy

## Abstract

*Streptococcus pneumoniae* is a major pathogen responsible for severe complications in patients with prior influenza A virus (IAV) infection. We have previously demonstrated that *S. pneumoniae* exhibits increased intracellular survival within IAV-infected cells. Fluoroquinolones (FQs) are widely used to treat pneumococcal infections. However, our prior work has shown that *S. pneumoniae* can develop intracellular FQ persistence, a phenomenon triggered by oxidative stress within host cells. This persistence allows the bacteria to withstand high FQ concentrations. In this study, we show that IAV infection enhances pneumococcal FQ persistence during intracellular survival within pneumocytes, macrophages, and neutrophils. This enhancement is partly due to increased oxidative stress induced by the viral infection. We find that this phenotype is particularly pronounced in autophagy-proficient host cells, potentially resulting from IAV-induced blockage of autophagosome-lysosome fusion. Moreover, we identified several *S. pneumoniae* genes involved in oxidative stress response that contribute to FQ persistence, including *sodA* (superoxide dismutase), *clpL* (chaperone), *nrdH* (glutaredoxin), and *psaB* (Mn^+2^ transporter component). Our findings reveal a novel mechanism of antibiotic persistence promoted by viral infection within host cells. This underscores the importance of considering this phenomenon when using FQs to treat pneumococcal infections, especially in patients with concurrent influenza A infection.

## Introduction

*Streptococcus pneumoniae* (pneumococcus), a bacterium colonizing the human nasopharynx, is a major cause of diverse infections, ranging from mild otitis media (ear infection) and sinusitis (sinus infection) to life-threatening meningitis and community-acquired pneumonia (CAP) (Anderson and Feldman, 2023). These severe pneumococcal infections significantly impact global health, causing over 1 million deaths annually (Walker et al., 2013; Wahl et al., 2018).

It is known that pneumococcal infections may result in severe pneumonia in patients previously infected with respiratory viruses such as influenza A, rhinovirus, adenovirus, metapneumovirus, respiratory syncytial virus, and SARS-CoV-2 (Westblade et al., 2021; Wada et al., 2024). These viral infections alter the immune response and allow secondary bacterial infections that are related to worse patient outcomes, particularly in those with comorbidities such as chronic respiratory and cardiovascular diseases, and diabetes mellitus (Cilloniz et al., 2022). We focused our study on the synergism that exists between *S. pneumoniae* and influenza A, the best viral/bacterial interaction described so far. *S. pneumoniae* is a notorious culprit in bacterial coinfections following influenza infection (Sender et al., 2021). Historical evidence from influenza outbreaks underscores how influenza significantly increases vulnerability to secondary bacterial infections (Smith and McCullers, 2014). Notably, coinfections involving both influenza virus and bacteria are far more severe and deadly than bacterial pneumonia alone (Joseph et al., 2013). Indeed, estimates suggest that secondary bacterial infections caused by *S. pneumoniae* may have been responsible for the majority of the 40–50 million deaths during the devastating 1918 Spanish influenza pandemic (Morens et al., 2008).

Influenza A virus (IAV) infection paves the way for secondary bacterial infections through a multi-pronged attack on the host’s defenses (McCullers, 2014). Firstly, the virus’s reliance on host cell machinery leads to widespread cellular dysfunction. IAV damages the lung’s epithelial lining, impairing mucociliary clearance, the body’s primary mechanism for expelling bacteria-laden mucus (McCullers, 2006). Additionally, viral neuraminidase alters the composition of mucus, creating a more hospitable environment for bacterial adhesion (McCullers, 2014). Secondly, IAV infection directly compromises immune cells, hindering their ability to engulf and destroy bacteria (McNamee and Harmsen, 2006). Finally, the virus triggers oxidative stress, overwhelming the delicate antioxidant balance within host cells. This imbalance weakens cellular defenses, creating an ideal environment for opportunistic bacteria to thrive.

During the infection process, *S. pneumoniae* employs a multifaceted strategy involving reactive oxygen species (ROS). This bacterium can produce ROS to gain a competitive advantage over resident microbiota in the respiratory tract, inflict damage on host cells (Ercoli et al., 2018), and disrupt inflammasome-dependent innate immune responses (Ogawa et al., 2018). Paradoxically, ROS are also critical components of the host’s defense mechanisms, posing a challenge that *S. pneumoniae* must overcome to thrive within host cells (Hernandez-Morfa et al., 2023). Intriguingly, research demonstrates that *S. pneumoniae* can survive within diverse host cell types, including pneumocytes (Cortes et al., 2015; Pinas et al., 2018; Reinoso-Vizcaino et al., 2020), macrophages (Cortes et al., 2015; Ercoli et al., 2018), and neutrophils (Martner et al., 2008; Barbuti et al., 2010), all of which generate varying levels of ROS. This remarkable intracellular persistence is facilitated by the bacterium’s ability to mount its own oxidative stress response, allowing it to tolerate both the ROS it produces and those released by the host’s immune cells (Yesilkaya et al., 2013; Li et al., 2015; Hernandez-Morfa et al., 2022, 2023). Our previous research demonstrated that *S. pneumoniae* can persist for extended periods within A549 pneumocytes, a process dependent on the bacteria’s oxidative stress response mechanisms (Pinas et al., 2018). Intriguingly, coinfection with IAV significantly enhances bacterial survival within these host cells. This synergistic effect is mediated by the pneumococcal SirRH two-component system, which regulates genes involved in both acid and oxidative stress tolerance (Reinoso-Vizcaino et al., 2020).

The global spread of antibiotic-resistant *S. pneumoniae* strains severely complicates treatment of invasive diseases like meningitis, a challenge worsened by IAV coinfection (MacIntyre et al., 2018; Feldman and Anderson, 2020). Fluoroquinolones (FQs) are critical for treating these infections, but their effectiveness is threatened by the emergence of FQ-resistant strains (Kim L. et al., 2016). Beyond resistance, bacteria such as *S. pneumoniae* employ adaptive mechanisms, including tolerance and persistence, to endure the presence of antibiotics. Unlike antibiotic resistance, tolerance and persistence are not genetically inherited traits, enabling bacteria to withstand lethal antibiotic levels without undergoing replication. While tolerance impacts the entire bacterial population, persistence is observed in only a specific subset (Balaban et al., 2019). Our work shows that oxidative stress encountered during host cell infection triggers FQ persistence in *S. pneumoniae*, affecting macrophages, neutrophils, and pneumocytes. This classifies it as a triggered persistence mechanism (Balaban et al., 2019). Furthermore, we have demonstrated that oxidative stress and growth arrest act synergistically to induce this FQ persistence in *S. pneumoniae* (Hernandez-Morfa et al., 2022, 2023).

This study investigates how IAV infection synergistically enhances FQ persistence in *S. pneumoniae* during intracellular survival in host cells. Our results demonstrate that expression of oxidative stress response genes in *S. pneumoniae* contributes to this influenza-induced persistence enhancement. By elucidating the interplay between IAV and pneumococcal oxidative stress mechanisms, we provide new insights into FQ persistence. This heightened persistence has potential clinical implications, suggesting that fluoroquinolone treatment of pneumococcal infections might be less effective in patients with concurrent influenza A infection.

## Results

### Influenza A coinfection increases *Streptococcus pneumoniae* survival in pneumocytes, but not in macrophages or neutrophils

**Figure 1:**
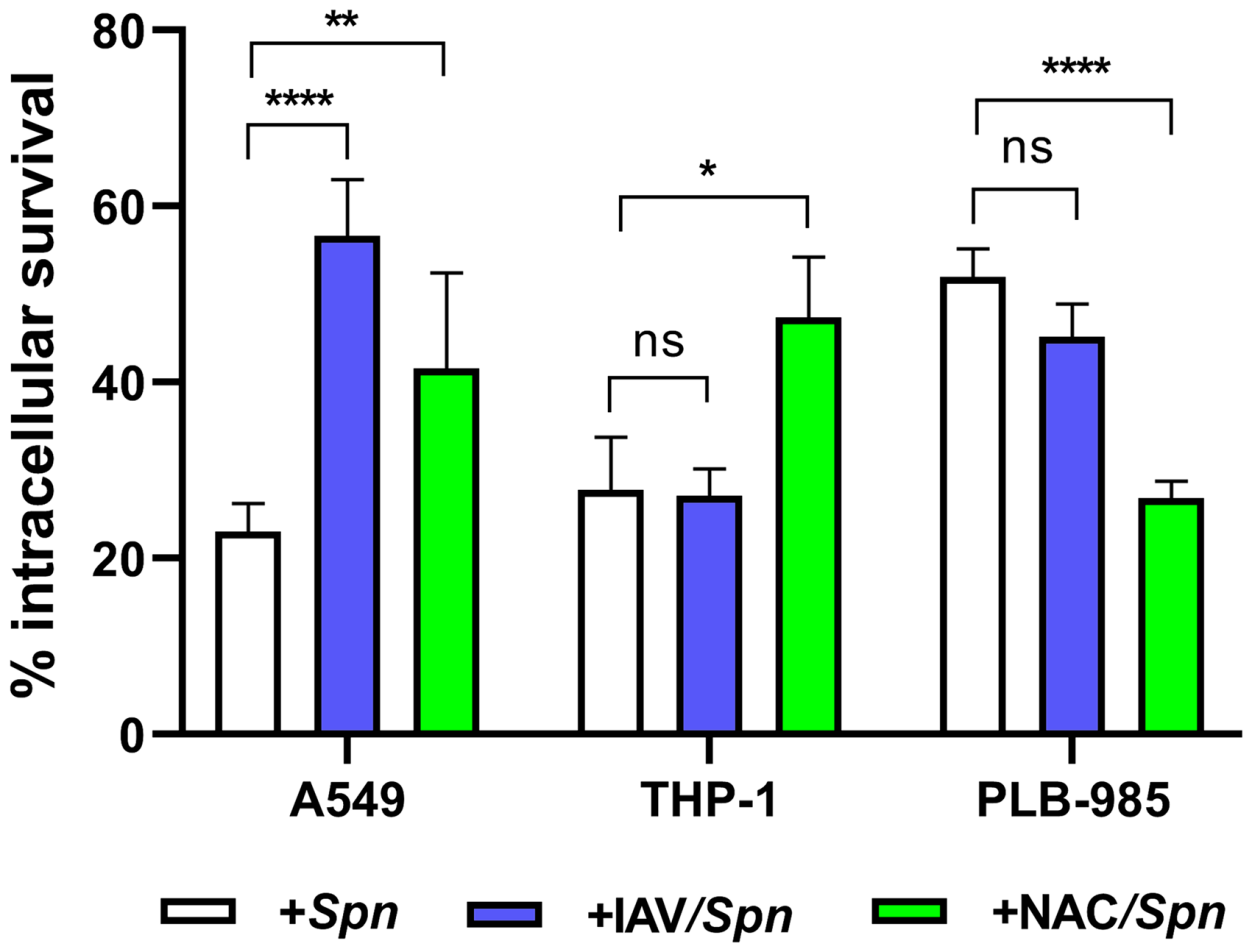
Synergistic increase in bacterial survival after IAV infection is specific to pneumocytes. A549 (lung epithelial), THP-1 (macrophages) and PLB-985 (neutrophils) cells were infected with IAV at a multiplicity of infection (MOI) of 10:1 for 24 h, followed by *S. pneumoniae* strain R801 (MOI 30:1). A549 cells were pre-treated (1 h) and co-treated (during bacterial infection) with 5 mM NAC, while PLB-985 and THP-1 cells received 10 mM NAC using the same regimen. The A549 and THP-1 cells were infected with pneumococci at a multiplicity of infection (MOI) of 30 for 3 h, and PLB-985 cells with the same MOI for 1 h. Bacterial survival was assessed using a gentamicin protection assay. In these assays, the 100% value of intracellular survival corresponds to the colony-forming units (CFUs) obtained after the gentamicin treatment, which was used to eliminate extracellular bacteria. Samples were collected at designated time points, lysed, diluted, and plated on BHI blood agar for CFU enumeration. Percentage survival represents the mean ± SEM of at least three replicates with statistically significant differences determined by a two-tailed test (**p < 0.01; ***p < 0.001; ****p < 0.0001). NS indicates non-significant. Data are representative of at least three independent experiments.

In a previous work, we have demonstrated that IAV coinfection increases *S. pneumoniae* survival within host cells by two main reasons. Firstly, the fusion between lysosomes and autophagosomes, where *S. pneumoniae* resides, is inhibited by the viral infection. In consequence, pneumococci are not killed by lysosomal enzymes and survive longer in autophagosomes. Secondly, in response to the heightened ROS production induced by IAV in host cells, pneumococci are compelled to upregulate the expression of various antioxidant proteins (such as SodA, TpxD, among others)(Reinoso-Vizcaino et al., 2020). In addition, we have reported that host cell oxidative stress induces pneumococcal FQ persistence (Hernandez-Morfa et al., 2022). We sought to determine whether IAV coinfection also enhances FQ persistence in human cell lines: A549 pneumocytes, THP-1 macrophages, and PLB-985 neutrophils. Building on our earlier findings in A549 cells (Reinoso-Vizcaino et al., 2020), we investigated this viral/bacterial synergy in THP-1 and PLB-985 cells using established infection protocols (Hernandez-Morfa et al., 2023). The *S. pneumoniae* R801 strain was incubated at a multiplicity of infection (MOI) of 30 for 3 h in A549 and THP-1 cells and for 1 h in PLB-985 cells. In viral/bacterial coinfections, host cells were first inoculated with the PR8 strain of IAV at a MOI of 10 for 24 h prior to bacterial inoculation, and the viral percentage (96–99%) was determined by flow cytometry (Supplementary Figure S1). Interestingly, while IAV coinfection reduced *S. pneumoniae* survival slightly in PLB-985 cells and showed no effect in THP-1 cells, it significantly increased bacterial survival within A549 pneumocytes (Figure 1). This cell-type specificity aligns with previous observations in 16HBE14o- bronchial cells (Reinoso-Vizcaino et al., 2020), and suggests a crucial difference in how pulmonary epithelial cells and immune cells handle IAV/pneumococcal coinfection. Our findings indicated that coinfection with influenza A enhances the survival of *S. pneumoniae* in pneumocytes, while no significant impact was observed in macrophages or neutrophils. Naturally, the robust antimicrobial mechanisms of immune cells likely explain their ability to better control intracellular pneumococcal survival compared to pulmonary cells.

Studies have shown that this viral infection increases ROS production in A549 pneumocytes (Komaravelli and Casola, 2014; Pyo et al., 2014). In our hands, the IAV infection of A549 pneumocytes augmented 33% ROS after 24 h (Reinoso-Vizcaino et al., 2020). To investigate the role of host cell oxidative stress in viral/bacterial synergy, we treated coinfected cells with N-acetylcysteine (NAC), a known inhibitor of ROS production. We used 5 mM NAC for A549 cells and 10 mM NAC for THP-1 and PLB-985 cells, applying the treatment starting 1 h before and during bacterial infection. Reducing ROS levels by the NAC treatment enhanced intracellular *S. pneumoniae* survival in both A549 and THP-1 cells (Figure 1), consistent with previous observations (Pinas et al., 2018). In contrast, PLB-985 cells exhibited reduced bacterial viability, potentially attributed to other ROS-independent NAC effect that improve phagocytic functions, as previously outlined (Oddera et al., 1994).

These results further support the critical role of ROS in regulating pneumococcal survival within host cells, consistent with our previous findings (Reinoso-Vizcaino et al., 2020). These results enable us to suggest that the induction of intracellular survival of *S. pneumoniae* is contingent upon the specific human cell line used in the coinfection assays.

### IAV boosts *Streptococcus pneumoniae* fluoroquinolone persistence

**Figure 2:**
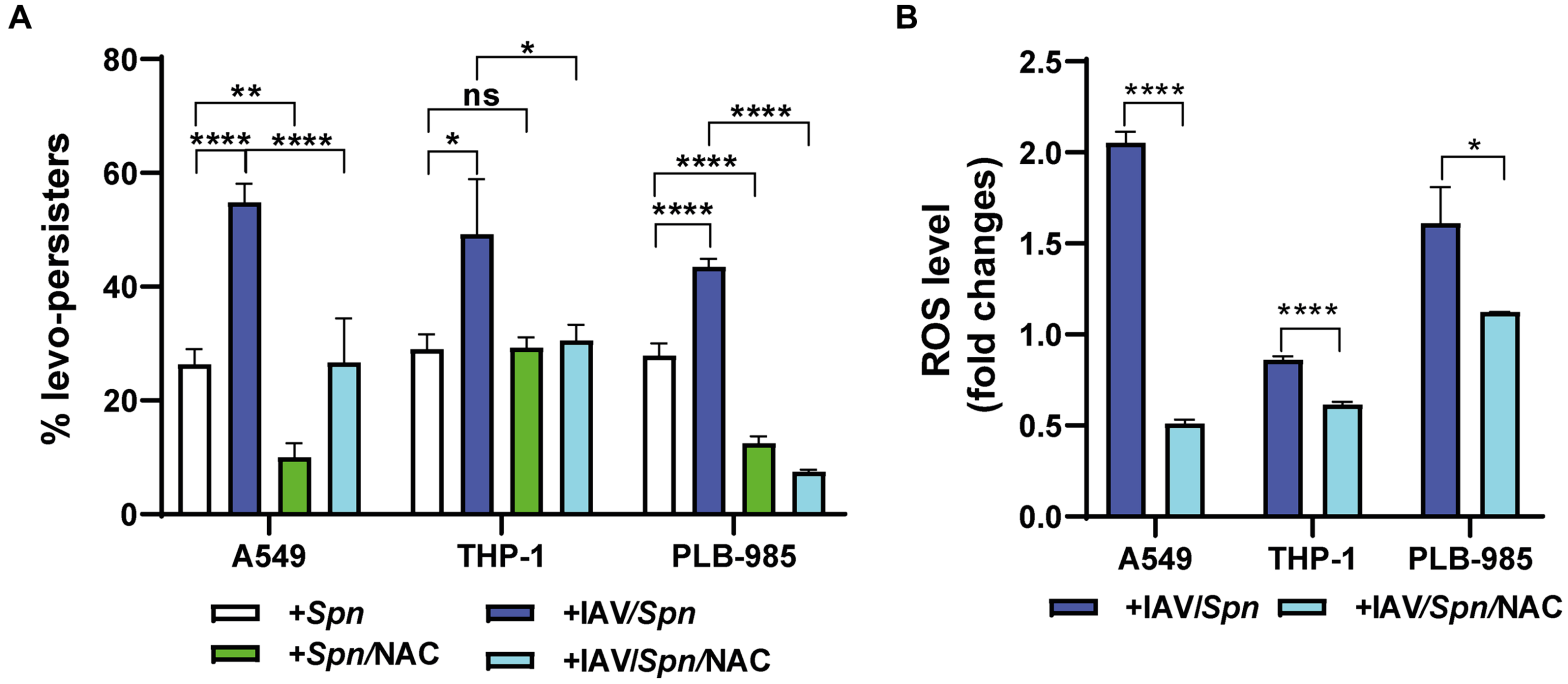
Influenza A Virus (IAV)-induced oxidative stress in host cells enhances levofloxacin persistence of *S. pneumoniae*. **(A)** Determination of Levofloxacin Persistence in Host Cells. A549, THP-1, and PLB-985 cells were infected with IAV at a MOI of 10:1 for 24 h. Subsequently, these cells were infected with pneumococci at a MOI of 30:1 using the same infection scheme shown in Figure 1. To assess the impact of ROS production, NAC treatment was performed as described in the Figure 1 legend. Bacterial survival was evaluated using a standard gentamicin protection assay. The percentage of levofloxacin-persisting bacteria was calculated relative to the total number of internalized bacteria. After the gentamycin treatment, which was used to eliminate extracellular bacteria, 6 μg/mL levofloxacin was added, and samples were collected at various time points depending on the cell type. Finally, the cells were lysed via centrifugation. Diluted samples in brain heart infusion (BHI) were plated on BHI blood agar and incubated for 16 h at 37°C. **(B)** Determination of ROS levels by flow cytometry. Intracellular ROS levels were quantified using the peroxide-sensitive fluorescent probe 2′,7′-dichlorodihydrofluorescein diacetate (H_2_DCFDA) as detailed in the Materials and Methods section. Fold changes represent the ratio of fluorescence intensity in IAV-infected cells compared to non-infected cells, with or without NAC treatment (as described in Figure 1 legend). Error bars represent the standard deviation of at least three replicates. Statistical significance was determined using a two-tailed t-test. Data are representative of at least three independent experiments, with statistically significant differences indicated as *p < 0.01, **p < 0.001, ***p < 0.0001.

Building upon the observed IAV-induced increase in *S. pneumoniae* intracellular survival in A549 pneumocytes, we investigated whether the virus also influences pneumococcal FQ persistence within host cells. Using our coinfection model, we exposed infected A549 cells, THP-1 macrophages, and PLB-985 neutrophils to levofloxacin (6 μg/mL). Intriguingly, we observed a significant increase in the proportion of levofloxacin persisters, hereafter, referred as levo- persisters (Figure 2A; Supplementary Figure S2). This indicates that viral infection promotes FQ persistence in *S. pneumoniae* across a range of cell types.

### Host cell oxidative stress induced by IAV infection promotes *Streptococcus pneumoniae* FQ persistence

We have previously shown that oxidative stress within host cells induces FQ persistence in *S. pneumoniae* (Hernandez-Morfa et al., 2022) and that IAV infection elevates intracellular ROS levels, aligning with other studies (Gennaris and Collet, 2013; Komaravelli and Casola, 2014). To directly test the link between ROS and FQ persistence, we treated cells with NAC for 1 h at either 5 mM (A549 cells) or 10 mM (THP-1 and PLB-985 cells), prior to bacterial challenge and followed by levofloxacin (6 μg/mL) treatment during bacterial infection. We observed a significant decrease in FQ persistence in *S. pneumoniae*-infected A549 and PLB-985 cells treated with NAC, confirming our previous findings (Hernandez-Morfa et al., 2022; Figure 2A). Importantly, in IAV-infected cells across all three cell types, NAC treatment similarly reduced *S. pneumoniae* FQ persistence (Figure 2A). This strengthens the connection between oxidative stress and FQ persistence and demonstrates that this mechanism remains relevant even during IAV coinfection.

Previous studies, including our own, have shown that IAV infection increases ROS production in A549 pneumocytes (Pyo et al., 2014; Reinoso-Vizcaino et al., 2020). Even during coinfection with IAV/*S. pneumoniae*, we confirmed this effect in A549, THP-1, and PLB-985 cells, demonstrating a significant fold-change increase in intracellular ROS levels upon IAV infection (Figure 2B). Importantly, pre-treating these IAV-infected cells with NAC (5 mM for A549, 10 mM for PLB-985 and THP-1 cells) for 1 h prior to bacterial challenge reduced both ROS levels and FQ persistence when measuring these phenotypes simultaneously (Figure 2B). This finding directly links IAV-induced oxidative stress to the heightened FQ persistence in *S. pneumoniae*. Despite this complexity, our data collectively indicates a strong association between IAV-induced oxidative stress in host cells and the heightened FQ persistence observed in *S. pneumoniae*.

### *Streptococcus pneumoniae* oxidative stress genes linked to FQ persistence during coinfection

From our previous work demonstrating the crucial role of SirRH in promoting *S. pneumoniae* survival during coinfection with IAV (Reinoso-Vizcaino et al., 2020), we sought to elucidate the underlying mechanisms. Transcriptomic and proteomic analyses revealed a link between SirRH and the expression of *clpL*, *nrdH*, and *psaB* genes under acidic conditions, which mimics the environment found within autophagosomes where *S. pneumoniae* resides during coinfection (Reinoso-Vizcaino et al., 2020). Interestingly, these genes are also potentially involved in the pneumococcal response to oxidative stress, as previously described for *psaB* that encodes a subunit of the Mn^2+^ transporter (McAllister et al., 2004; McCluskey et al., 2004) and *sodA* that encodes a superoxide dismutase (not regulated by SirR) (Eijkelkamp et al., 2014). We hypothesized that this network contributes to the complex phenomenon of fluoroquinolone (FQ) persistence.

**Figure 3:**
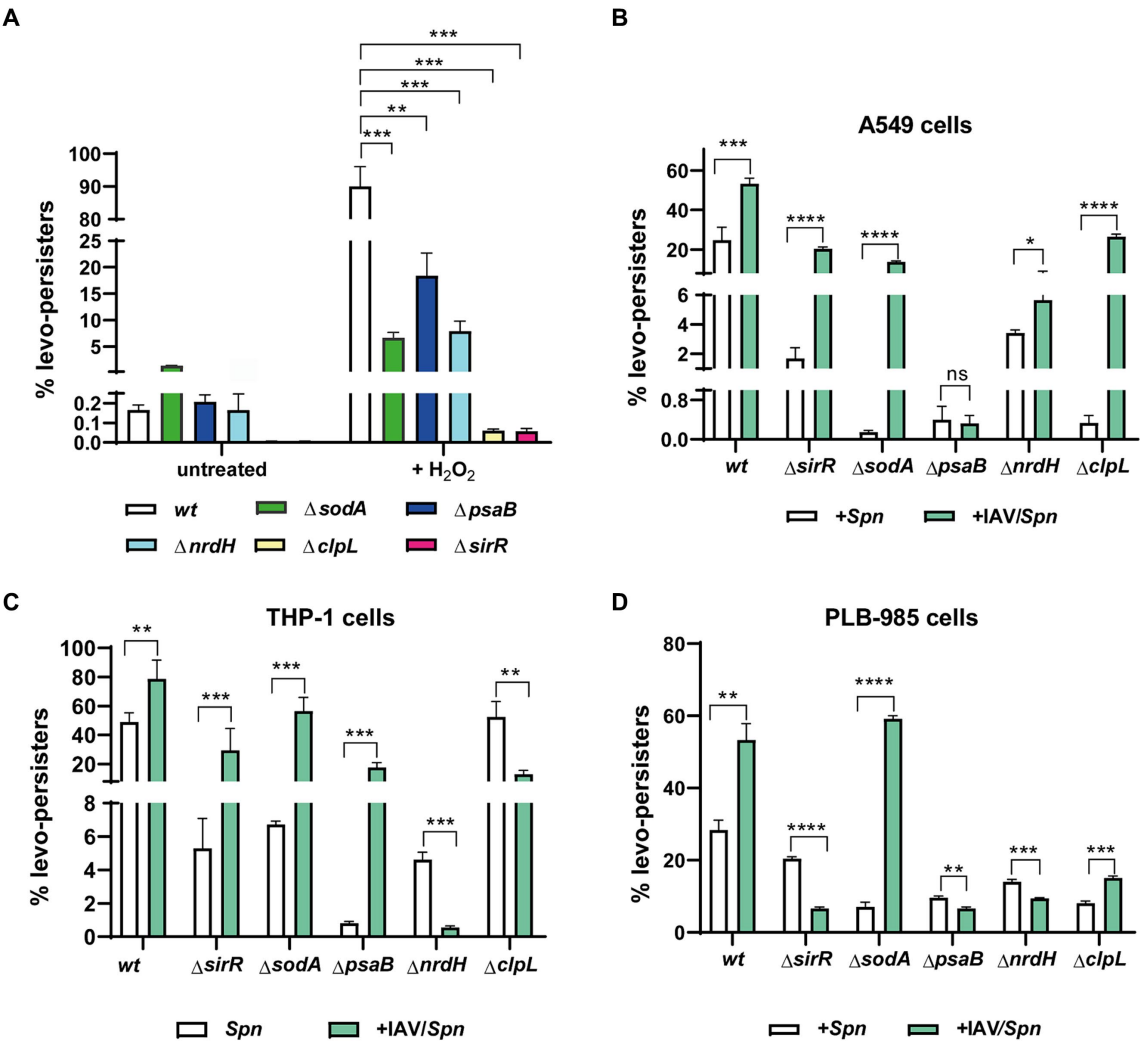
Involvement of *clpL, sodA, psaB, nrdH*, and *sirR* genes in levofloxacin persistence during *S. pneumoniae* infection in host cells. **(A)** Decreased FQ persistence of *ΔclpL, ΔsodA, ΔpsaB, ΔnrdH*, and *ΔsirR* mutants in culture media. *S. pneumoniae* strains (*wt* and indicated mutants) were grown to mid-exponential phase (OD_600nm_ 0.3) and exposed to oxidative stress (20 mM H_2_O_2_ for 30 min) or left untreated. Following stress/no-stress treatment, cultures were exposed to 6 μg/mL levofloxacin, and FQ persistence was evaluated as described previously. **(B–D)** Influence of IAV infection on FQ persistence in *ΔclpL, ΔsodA, ΔpsaB, ΔnrdH*, and *ΔsirR* mutants within A549, THP-1, and PLB-985 cells. FQ persistence of each pneumococcal mutant was analyzed in both IAV-infected and non-infected cells of the indicated host cell line. Experimental details for infection and FQ persistence assays are provided in the Materials and Methods section and Figure 2. Error bars represent the standard deviation of at least three replicates. Statistical significance was determined using a two-tailed *t*-test, indicated as *p < 0.01, **p < 0.001, or ****p < 0.0001.

To investigate this hypothesis, we systematically generated *S. pneumoniae* mutants lacking each of these genes (*clpL, nrdH, psaB*, and *sodA*) (Supplementary Table S1). As anticipated, all mutants showed increased susceptibility to H_2_O_2_-induced oxidative stress (Supplementary Figure S3), confirming their importance in combating oxidative damage. And following exposure to H_2_O_2_ (20 mM) and subsequent treatment with levofloxacin (6 μg/mL), a significant decrease in FQ persistence was observed for all mutants compared to wild-type pneumococci (Figure 3A; Supplementary Figure S4). This suggests these genes contribute to an intrinsic stress tolerance mechanism in *S. pneumoniae*, one that likely helps pneumococci withstand antibiotic pressure even in the absence of host cell interactions.

In IAV-infected A549 pneumocytes, THP-1 macrophages, and PLB-985 neutrophils, mutants generally displayed decreased FQ persistence across all cell lines compared to the wild-type strain (Figures 3B–D; Supplementary Figures S5–S7). This highlights the intriguing possibility that IAV infection promotes FQ persistence in *S. pneumoniae* by activating pathways that inadvertently increase antibiotic tolerance. We speculate that this might occur through IAV-triggered, SirRH-mediated pneumococcal stress response pathways involving these genes.

However, this relationship becomes more nuanced when we compare mutant behavior between different cell types (Figure 3). The unique behavior of the *ΔclpL* mutant in THP-1 cells (no effect on FQ persistence, Figure 3C) underscores that cell-type specific factors likely modulate FQ persistence during coinfection. Similarly, despite exhibiting reduced FQ persistence in single-infection models, IAV coinfection generally increased FQ persistence in most mutants across A549 and THP-1 cells, with notable exceptions: *ΔpsaB* in A549 (Figure 3B) and *ΔnrdH* & *ΔclpL* in THP-1 (Figure 3C). Conversely, most mutants in coinfected PLB-985 cells showed decreased FQ persistence, except *ΔsodA* and *ΔclpL* (Figure 3D). These findings paint a complex picture. While IAV infection seems to generally promote FQ persistence through oxidative stress pathways in *S. pneumoniae*, the specific genes involved, and the magnitude of the effect appear to be highly dependent on the oxidative environment and unique defense mechanisms of different host cell types.

### Autophagy in host cells is essential for IAV-induced FQ persistence in *Streptococcus pneumoniae*

**Figure 4:**
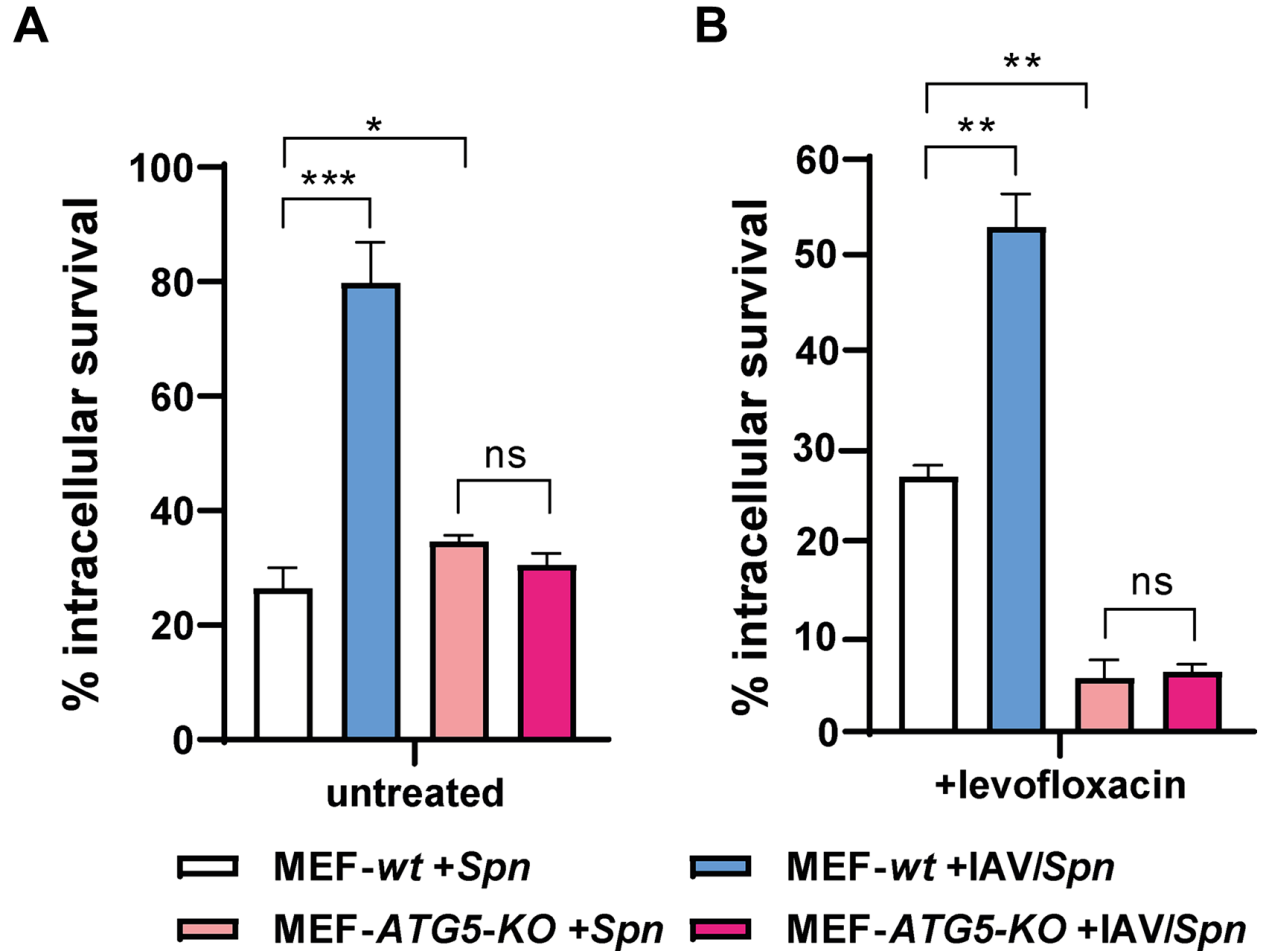
Autophagy-proficient MEFs exhibit Levofloxacin persistence. Wild-type MEFs (MEF *wt*) and autophagy-deficient MEFs lacking ATG5 (MEF *ATG5-KO*) were infected with IAV at a MOI of 10:1. After 24 h, the cells were co-infected with *S. pneumoniae* for 3 h. Intracellular pneumococcal survival was assessed with standard gentamicin protection assay. The percentage of surviving *S. pneumoniae* was determined relative to the total internalized bacteria after 30 min of gentamicin treatment (considered 100%). To quantify levofloxacin-persistent bacteria (levo-persisters), cells were exposed to 6 μg/mL levofloxacin immediately after gentamicin treatment, followed by lysis and plating on BHI blood agar plates after 3 h. The data represent the mean with standard deviation from three independent samples. Error bars represent the standard deviation of at least three replicates. Statistical significance was determined using a two-tailed t-test. Data are representative of at least three independent experiments, with statistically significant differences indicated as *p < 0.01, **p < 0.001, ***p < 0.0001.

We had established that influenza A virus (IAV) promotes *S. pneumoniae* intracellular survival via a mechanism dependent on host cell autophagy (Reinoso-Vizcaino et al., 2020). To delve deeper into the relationship between autophagy and IAV-induced FQ persistence, we utilized a coinfection model with mouse embryonic fibroblasts (MEFs). Specifically, we compared autophagy-deficient MEF *ATG5-KO* cells (which lack the crucial Atg5 protein) to their autophagy-proficient wildtype counterparts (MEF-*wt*) (Kuma et al., 2004; Nishida et al., 2009; Zhou et al., 2009; Don Wai Luu et al., 2022). Confirming our earlier findings, IAV coinfection failed to enhance *S. pneumoniae* survival in MEF *ATG5-KO* cells (Figure 4; Supplementary Figure S8), underscoring the importance of autophagy in this process (Reinoso-Vizcaino et al., 2020).

Next, we investigated levofloxacin persistence. Intriguingly, we observed the expected IAV-induced increase in FQ persistence only within the autophagy-proficient MEF-*wt* cells (Figure 4; Supplementary Figure S8), paralleling our observations in human cell lines. Critically, in MEF *ATG5-KO* cells, where autophagy is impaired, FQ persistence actually decreased regardless of IAV infection. These results provide compelling evidence that IAV-induced FQ persistence in *S. pneumoniae* is directly linked to functional autophagy pathways within host cells. This exciting finding opens avenues for further investigation into the precise mechanisms by which autophagy contributes to this antibiotic persistence phenomenon.

**Figure 5:**
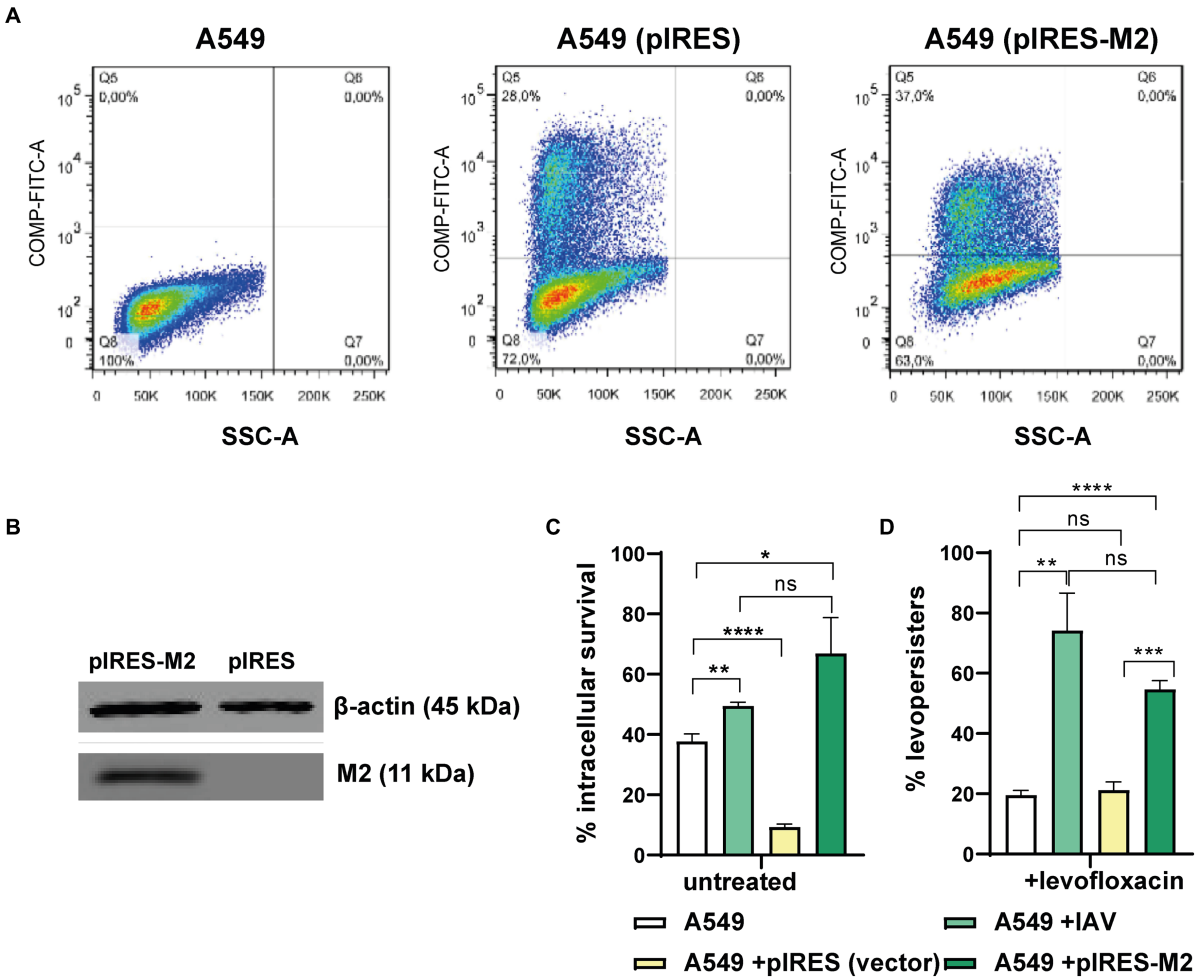
Viral M2 protein and levo-persistence. **(A)** Transfection Efficiency: A549 cells were transfected with pIRES (empty vector) or pIRES-M2 plasmids. Transfection efficacy was evaluated using GFP expression (a fluorescent marker within the pIRES vector) by flow cytometry. Green fluorescence intensity (FITC-A channel) is shown for non-transfected, pIRES-transfected, and pIRES-M2-transfected cells. The percentage of transfected cells is displayed in the Q5 quadrant of each graph. **(B)** M2 Protein Expression: Western blot analysis confirmed M2 protein expression in A549 cells transfected with pIRES-M2 compared to control (pIRES). β-actin served as a loading control. **(C)** Levo-persistence and M2: *S. pneumoniae* persistence was assessed in A549 cells transfected with pIRES or pIRES-M2, followed by bacterial infection. For comparison, A549 cells were infected with IAV before bacterial infection to mimic M2 expression during viral infection. Intracellular bacterial survival was measured using a gentamicin protection assay. Levo-persistence was determined following levofloxacin treatment as described in Figure 3. Data represent at least three independent experiments. Statistical significance is indicated: *p < 0.01, **p < 0.001, ****p < 0.0001.

Based on previous data showing that IAV M2 protein blocks autophagosome-lysosome fusion (Gannage et al., 2009), and that IAV promotes *S. pneumoniae* survival within autophagosomes (Reinoso-Vizcaino et al., 2020), we hypothesized that M2 could independently drive both intracellular survival and FQ persistence. To test this, we expressed M2 protein in A549 pneumocytes using a vector that also produces a green fluorescent protein (GFP) marker (pIRES-M2). Transfection efficiency was confirmed by flow cytometry (37%, Figure 5A; Supplementary Table S1). Western blot analysis verified M2 protein expression only in pIRES-M2 transfected cells and not in control cells transfected with an empty vector (Figure 5B). Critically, A549 cells expressing M2 displayed increased *S. pneumoniae* survival and FQ persistence, mirroring the effects observed in IAV-infected cells (Figure 5C). Conversely, control cells transfected with the empty vector showed survival rates like non-infected cells. These findings strongly suggest that the influenza A M2 protein alone is sufficient to recapitulate the IAV-mediated enhancement of both *S. pneumoniae* intracellular survival and FQ persistence within A549 pneumocytes.

## Discussion

*Streptococcus pneumoniae* and IAV are major human pathogens (McCullers, 2014). *S. pneumoniae* causes invasive disease with high mortality (55% in children with pneumonia) in low-and middle-income countries, contributing to over 1.5 million deaths globally (GBD-LRI, 2017). IAV infections result in roughly 1 billion cases, 3–5 million severe illnesses, and 300,000–500,000 deaths worldwide annually (Klein et al., 2016). Influenza A infections frequently become more severe due to secondary bacterial infections, especially those caused by *S. pneumoniae*. This co-infection worsens respiratory problems and significantly increases illness and death rates (Joseph et al., 2013; Smith and McCullers, 2014; Cilloniz et al., 2022; Okahashi et al., 2022). Estimates suggest a substantial portion of the 40–50 million deaths during the 1918 Spanish influenza pandemic stemmed from secondary pneumococcal infections (Brundage and Shanks, 2008; Morens et al., 2008). Similarly, around 34% of fatalities attributed to the 2009 influenza A pandemic involved subsequent bacterial infections, with *S. pneumoniae* being the most common culprit (Dawood et al., 2012; Chertow and Memoli, 2013). Recent studies have shown that in controlled environments, 11–35% of confirmed influenza cases were linked to secondary pneumococcal infections (Klein et al., 2016). This situation presents a substantial treatment challenge, as it raises the risk of ineffective initial antibiotic therapy and poorer patient outcomes. The growing problem of antimicrobial resistance makes managing *S. pneumoniae* infections with antibiotics increasingly difficult (Reinert, 2009; Feldman and Anderson, 2020; Zahari et al., 2023). In recognition of this, the World Health Organization’s Global Antimicrobial Resistance Surveillance System (GLASS) has identified *S. pneumoniae* as a priority pathogen for antibiotic-resistant surveillance (GLASS, 2015).

Fluoroquinolones (FQs) are widely used to treat invasive pneumococcal disease. However, the emergence of FQ-resistant strains compromises their effectiveness (Kim T. et al., 2016). Beyond resistance, bacterial persistence poses an additional challenge. Persisters are a phenotypic subpopulation that can tolerate high, typically lethal antibiotic concentrations without altering the minimum inhibitory concentration (Gefen and Balaban, 2009; Kester and Fortune, 2014).

Our group previously identified the first antibiotic persistence mechanism in *S. pneumoniae* triggered by oxidative stress during intracellular survival within host cells. This mechanism allows the bacteria to tolerate lethal FQ concentrations (Hernandez-Morfa et al., 2022, 2023). In this study, we demonstrate that coinfection of host cells with IAV and *S. pneumoniae* increases bacterial FQ persistence. This phenomenon is observed across human pneumocytes, macrophages, and neutrophils, suggesting a cell-type independent mechanism (Hernandez-Morfa et al., 2022, 2023).

Initially, we proposed two hypotheses to explain this response to viral coinfection and that impact on FQ persistence. The first one posits that IAV infection enhances intracellular survival of pneumococci into pneumocytes, thereby increasing FQ persistence.

*S. pneumoniae*’s ability to persist within splenic macrophages suggests a potential reservoir for septicemia (Ercoli et al., 2018). Moreover, evidence demonstrates pneumococci residing in autophagic vesicles during early infection stages in human nasopharyngeal epithelial cells and mouse embryonic fibroblasts (Ogawa et al., 2018). Our research further indicates that *S. pneumoniae*’s stress response mechanisms promote its survival within A549 pneumocytes (Cortes et al., 2015; Pinas et al., 2018). This synergistic effect requires functional autophagy pathways and correlates with pneumococcal survival inside autophagosomes (Reinoso-Vizcaino et al., 2020). IAV infection blocks lysosomal fusion via its M2 protein, leading to autophagosome accumulation (Gannage et al., 2009). We therefore hypothesized that M2 contributes to the observed increase in intracellular *S. pneumoniae* survival during co-infection (Reinoso-Vizcaino et al., 2020). Accordingly, in this work we found that expressing the M2 protein in pneumococci infected A549 pneumocytes significantly increased *S. pneumoniae* intracellular survival, similar to the effect observed during IAV infection. Furthermore, M2 expression augmented FQ persistence. Given our previous findings that *S. pneumoniae* accumulates within autophagosomes in IAV-infected A549 cells (Reinoso-Vizcaino et al., 2020) and that FQ persistence is induced within autophagy-proficient cells, we propose that FQ persistence of *S. pneumoniae* in A549 and MEF cells infected by IAV is a consequence of bacterial survival within autophagosomes.

Apparently, the first hypothesis regarding the FQ persistence mechanism *of S. pneumoniae* in host cells coinfected with IAV, which involves an increased intracellular survival, is valid for pulmonary cells. However, this phenotype was not detected in macrophages, indicating the existence of another IAV-induced mechanism that increases FQ persistence in host cells. An alternative hypothesis to explain FQ persistence emphasizes the intracellular oxidative environment within host cells, particularly during pneumococcal-IAV coinfection. Prior studies indicate IAV infection increases host cell ROS production (Pyo et al., 2014). We propose this IAV-induced oxidative stress may trigger FQ persistence in *S. pneumoniae*. This hypothesis is supported by our observation that the antioxidant N-acetylcysteine (NAC) significantly reduced FQ persistence in all coinfected cell lines. Further, we detected elevated intracellular ROS during coinfection of A549 pneumocytes, THP-1 macrophages and PLB-985 neutrophils with influenza A. This confirms the relevance of the oxidative stress response on the induction of FQ persistence in host cells.

Our previous work identified *S. pneumoniae* oxidative stress genes (e.g., *spxB*, *sodA*, *tpxD*) involved in host cell ROS-induced FQ persistence. These genes, regulated by StkP-ComE signaling (Pinas et al., 2018), exhibited reduced FQ persistence upon mutation in A549, Raw 264.7, and PLB-985 infection models (Hernandez-Morfa et al., 2022). We further established the SirRH two-component system’s critical role in the cooperative effect of IAV infection on intracellular *S. pneumoniae* survival (Reinoso-Vizcaino et al., 2020). Comparative transcriptomic analysis revealed that the SirR response regulator governs the expression of oxidative stress genes, including *clpL* (encoding a chaperone), *psaB* (encoding an Mn^+2^ transporter), and *nrdH* (encoding a glutaredoxin-like protein) (Reinoso-Vizcaino et al., 2020). Mutations in *sirR*, *clpL*, *psaB*, and *nrdH* significantly reduced FQ persistence within host cells and diminished the response to oxidative stress compared to the wild-type strain (Reinoso-Vizcaino et al., 2020). Notably, the *ΔnrdH* mutant displayed a similar phenotype, highlighting a novel role for this gene in bacterial persistence. These findings corroborate our previous work demonstrating the importance of other oxidative stress genes like *spxB*, *tpxD*, and *sodA* (used as a control here) in FQ persistence (Hernandez-Morfa et al., 2022).

Here, we report the first characterization of a bacterial defense mechanism against antibiotics enhanced by intracellular survival within host cells due to a viral coinfection. The impact of mutations in genes associated with oxidative stress response (*sirR, sodA, clpL, psaB*, and *nrdH*) on FQ persistence was assessed. Coinfected A549 and THP-1 cells displayed enhanced FQ persistence in most mutants compared to pneumococci-only infections. However, this effect was less pronounced in PLB-985 cells, suggesting the influence of cell-type specific responses. This highlights the functional diversity of oxidative stress genes in *S. pneumoniae*, with distinct enzymes potentially playing varying roles in different host cell environments. These findings underscore the importance of a robust oxidative stress response for *S. pneumoniae* survival and FQ persistence within host cells. Furthermore, our data indicate that the synergistic effect of IAV coinfection on FQ persistence extends beyond just the oxidative stress response. Mechanisms such as M2-mediated blockage of autophagosome-lysosome fusion, previously reported to enhance intracellular survival (Gannage et al., 2009), likely also contribute.

Previously, we proposed that the induction of FQ persistence within host cells could impact the effectiveness of antibiotic treatment in patients with pneumococcal infections (Hernandez-Morfa et al., 2022). Importantly, persistence to beta-lactams and FQs have been reported in clinical strains of *S. pneumoniae* (Geerts et al., 2022). In this study, we particularized on the exacerbation of this phenomenon due to influenza A virus (IAV) infection, speculating that the use of FQs in coinfected individuals may complicate antimicrobial therapy. Furthermore, we posit that prolonged exposure to these antibiotics could facilitate the development of FQ resistance. This resistance is acquired through a gradual accumulation of genetic mutations in the quinolone resistance-determining regions (QRDRs) of the *parC* gene (encoding topoisomerase IV) and/or the *gyrA* gene (encoding DNA gyrase) (Kim L. et al., 2016).

In conclusion, this study shows that enhanced antibiotic persistence due to IAV co-infection could make treatment of *S. pneumoniae* infections more difficult. Prolonged antibiotic exposure of FQ persisters could potentially contribute to the development of full-fledged FQ resistance in *S. pneumoniae*. Thus, further research is needed to evaluate the emergence of FQ resistance among FQ persisters in *S. pneumoniae.*

## Materials and methods

### Bacterial strains, growth conditions, and primers

Bacterial strains, plasmids, and oligonucleotides used in this study are described in Supplementary Table S1. Oligonucleotide synthesis and DNA sequencing services were performed by Macrogen Inc. (Seoul, South Korea). The mutagenesis procedure (Echenique and Trombe, 2001), DNA transformation procedures (Cortes et al., 2008a), growth conditions (Cortes et al., 2008b), and stock preparation of the pneumococcal strains (Albarracin Orio et al., 2008) have been previously reported.

### Antibiotic survival assay

Pneumococcal strains were cultured in Brain Heart Infusion (BHI) media at 37°C until an optical density of 0.3 at 600 nm (OD_600nm_) was reached. To induce persister formation, cultures were exposed to 20 mM H_2_O_2_ for 30 min. Bacterial cells were then centrifuged, washed with PBS, and resuspended in fresh culture media. Following H_2_O_2_ treatment, cultures were exposed to 6 μg/mL levofloxacin for 5 h, in accordance with an established protocol (Hernandez-Morfa et al., 2022). Samples were collected after the 5-h incubation, serially diluted, and plated on blood agar plates for quantification. Surviving colonies were counted and reported as colony-forming units per milliliter (CFU/mL), representing FQ persisters. Minimum inhibitory concentrations (MICs) were determined using the broth microdilution method as recommended by the Clinical and Laboratory Standards Institute (CLSI) (CLSI, 2021).

### Preparation of influenza A virus stock

The influenza A/Puerto Rico/8/34 (H1N1) strain (PR8) was used for this study (Francis and Magill, 1937). Viral stocks were propagated in 9-day-old specific pathogen-free (SPF) embryonated chicken eggs (sourced from Avico, Argentina). Each egg was inoculated via the amniotic cavity with 200 μL of diluted viral sample. The dilutions were prepared in phosphate-buffered saline (PBS) supplemented with gentamicin to prevent bacterial contamination. Eggs were incubated for 72 h at 37°C. Following incubation, the allantoic fluid was harvested, aliquoted, and stored at −80°C. Madin-Darby canine kidney cells (MDCK; ATCC CCL-34) were used to determine the titer of the propagated viral stock (Chockalingam et al., 2012; Reinoso-Vizcaino et al., 2020).

### Cell lines and culture conditions

A549 cells (ATCC CCL-185, from human lung epithelial carcinoma) were cultured in DMEM (4.5 g/L glucose, 10% heat-inactivated FBS; Gibco BRL, Gaithersburg, MD) at 37°C, 5% CO_2_. Cells were passaged every 2–3 days using trypsin/EDTA (25 μg/mL, 0.5 mM) and seeded into 75 cm^2^ flasks (Greiner Bio-one) before transfection (Reinoso-Vizcaino et al., 2020). Transfections with pIRES2-EGFP and/or pIRES2-M2 used JetPRIME (Polyplus-transfection, Illkirch, France) in serum-free DMEM with 5% FBS (Reinoso-Vizcaino et al., 2020). Mouse Embryonic Fibroblast (MEF) and autophagy-deficient MEF *ATG5*-*KO* cell lines (Kuma et al., 2004) were generously provided by Dr. Noboru Mizushima (The University of Tokyo, Japan) and cultured under the same conditions as described for A549 cells. The human monomyelocytic PLB-985 and PLB-985 *gp91^phox^* KO (PLB-985-*KO*) cell lines (provided by Prof. Mary C. Dinauer, Indiana University, USA) (Zhen et al., 1993; Panday et al., 2015) were cultured in RPMI 1640 (1% penicillin/streptomycin, 10% heat-inactivated FBS) at 37°C, 5% CO_2_, with density maintained below 1.5 × 106 cells/mL (Zhen et al., 1993). The *gp91^phox^* gene (also known as CYBB or nox2) encodes the cytochrome β-245, β-chain NADPH oxidase 2 (gp91^phox^ or Nox2), the catalytic subunit of NADPH oxidase, a major source of cellular reactive oxygen species (ROS). THP-1 cells were grown in RPMI 1640 supplemented with 1% non-essential amino acids, 50 μM β-mercaptoethanol, 10% FBS (Sigma-Aldrich Co), and 1% penicillin/streptomycin. Macrophage differentiation used 500,000 cells/well with 50 ng/mL PMA for 24 h. All cell lines were routinely tested for *Mycoplasma, Acholeplasma*, and *Ureaplasma* (Shahhosseiny et al., 2010).

### Neutrophil differentiation by DMSO treatment

Neutrophil differentiation in PLB-985 and PLB-985-KO cells was induced as described previously (Hernandez-Morfa et al., 2022). Cells were seeded at 1×10^5^ cells/mL in RPMI 1640 supplemented with 5% FBS, 1% penicillin/streptomycin, and 1.3% DMSO (Sigma-Aldrich Co.) for 5 days to mimic neutrophil development (Rincon et al., 2018). Cell density was adjusted to 1×10^6 cells/mL on day 3 and maintained throughout differentiation. Undifferentiated PLB-985 cells were cultured without DMSO as controls. Flow cytometry analysis of CD11b surface expression confirmed successful differentiation, as reported previously (Pedruzzi et al., 2002).

### Influenza A infection of host cells

After cells were infected with IAV (MOI 10) for 24 h at 37°C, the viral infection was confirmed by targeting the HA protein of IAV. Cells were incubated with the anti-HA primary antibody (rabbit) for 1 h, as HA is a protein specific to IAV, followed by incubation with the secondary antibody labelled with Alexa Fluor 633 (goat, anti-rabbit, Invitrogen) for 30 min, using the protocol suggested by the manufacturer (Invitrogen). Finally, the samples were analyzed using the FACS Canto cytometer to detect the Alexa Fluor 633 signal and to quantify the percentage of IAV-infected cells.

### Determination of intracellular survival and FQ persistence in IAV/pneumococci coinfected cells

Intracellular pneumococcal survival assays followed established protocols (Hernandez-Morfa et al., 2022). A549, MEF, MEF-*ATG5-KO*, PLB-985, PLB-985-*KO* or THP-1 cells (1.5×10^5^/well) were seeded in 12-well plates (Greiner Bio-One) and incubated for 24 h in DMEM or RPMI (5% FBS) with cell line-specific supplements. Cells were then infected with IAV (MOI 10) for 24 h at 37°C. Antioxidant treatment used NAC (Sigma): 5 mM for A549, 10 mM for THP-1, MEF, MEF-ATG5-KO, and PLB-985 cells, applied 1 h before and during bacterial infection. The *ΔsodA, ΔpsaB, ΔsirR, ΔclpL, ΔnrdH*, and *wt S. pneumoniae* strains were grown in BHI to OD_600nm_ 0.3, then resuspended in cell-specific media (DMEM/RPMI, 5% FBS). Cells were infected at a bacterial MOI of 30 for 3 h (A549) or 1 h (THP-1, MEF, MEF-*ATG5-KO*). After washing with PBS, fresh media with 200 μg/mL gentamicin sulfate (US Biological) was added to eliminate extracellular bacteria. Optimization confirmed 30 min gentamicin exposure was sufficient (Cortes et al., 2015). Bacterial counts at this point were designated 100% survival for subsequent normalization. To assess FQ-persistence, infected A549, THP-1, MEF, or MEF-*ATG5-KO* cells were cultured in DMEM (1% FBS, 6 μg/mL levofloxacin) (Hernandez-Morfa et al., 2022). For suspension-grown PLB-985 cells, low-speed centrifugation (1,000 g) was used before 30 min gentamicin treatment. Cells were then cultured in RPMI (1.3% DMSO, 6 μg/mL levofloxacin) (Hernandez-Morfa et al., 2022). Intracellular bacteria were released by centrifugation (15,000 g, 5 min), resuspended in BHI, and plated on BHI-agar-blood for CFU counting. IAV and IAV/pneumococcus-induced apoptosis/necrosis was determined by flow cytometry (Annexin V/propidium iodide kit, Invitrogen).

### Quantification of ROS in host cells

Intracellular ROS accumulation in host cells was measured using the peroxide-sensitive fluorescent probe 2′,7′ dichloro-dihydrofluorescein diacetate (H_2_DCFDA; Sigma) (Satoh et al., 1996). A549, PLB-985, THP-1, and MEF cells were collected at designated time points and centrifuged (1,800 g). Cell pellets were PBS-washed and resuspended in PBS (GIBCO) with 10 μM dihydrofluorescein diacetate. After 30 min of dark incubation at 37°C, cells were centrifuged again and resuspended in PBS containing 50 μg/mL propidium iodide for dead cell detection. Flow cytometry (Beckton Dickinson FACSCanto II) analyzed DCF fluorescence distribution (excitation: 488 nm, emission: 535 nm). Mitochondrial ROS measurement followed a similar procedure, with the addition of a 10 μM dihydrofluorescein diacetate exposure step to assess total ROS after initial centrifugation. FlowJo™ v 7.6.2 Software (BD Life Sciences) was used for data analysis.

### Protein expression analysis by western blot

Western blot analysis was performed as described (Reinoso-Vizcaino et al., 2020). Briefly, A549 cells transfected with pIRES or pIRES-M2 were lysed with Laemmli solution (5% β-mercaptoethanol) and heated (90°C, 5 min). Supernatants were collected, and 40 μg of each sample was separated on 15% SDS-PAGE gels in Tris-Glycine-SDS running buffer (100 V cm-1). Proteins were transferred to PVDF membranes (wet transfer) and blocked with 5% BSA-PBS (1 h, room temperature). Membranes were incubated overnight at 4°C with primary antibodies against Influenza A M2 protein (ab5416, Abcam; 1:1000 dilution in 5% BSA-PBS) and β-actin (ab8226, Abcam; 1:2000 dilution). After washing (0.5% Tween-TBS), membranes were incubated with Alexa-conjugated secondary antibodies (1:1000 dilution, 1 h, room temperature). Fluorescence was detected using the Odyssey CLx Imaging System (LI-COR).

## Data availability statement

The original contributions presented in the study are included in the article/Supplementary material, further inquiries can be directed to the corresponding author.

## Ethics statement

Ethical approval was not required for the studies on humans and animals in accordance with the local legislation and institutional requirements because only commercially available established cell lines were used.

## Author contributions

MH-M: Conceptualization, Data curation, Formal analysis, Investigation, Methodology, Software, Writing – review & editing. NR-V: Formal analysis, Methodology, Supervision, Validation, Writing – review & editing. VZ: Formal analysis, Methodology, Writing – review & editing. NO: Investigation, Methodology, Writing – review & editing. PC: Investigation, Writing – review & editing. CS: Methodology, Writing – review & editing. DP: Funding acquisition, Writing – review & editing. JE: Conceptualization, Formal analysis, Funding acquisition, Investigation, Project administration, Resources, Supervision, Visualization, Writing – original draft, Writing – review & editing.
