# Supplementary material for: Intracellular Streptococcus pneumoniae develops enhanced fluoroquinolone persistence during influenza A coinfection
Source: Front Microbiol. 2024 Jul 5;15:1423995. doi: 10.3389/fmicb.2024.1423995 (PMC11258013; doi:10.3389/fmicb.2024.1423995)
Supplement: Supplementary file 1 [file Data_Sheet_1.PDF]

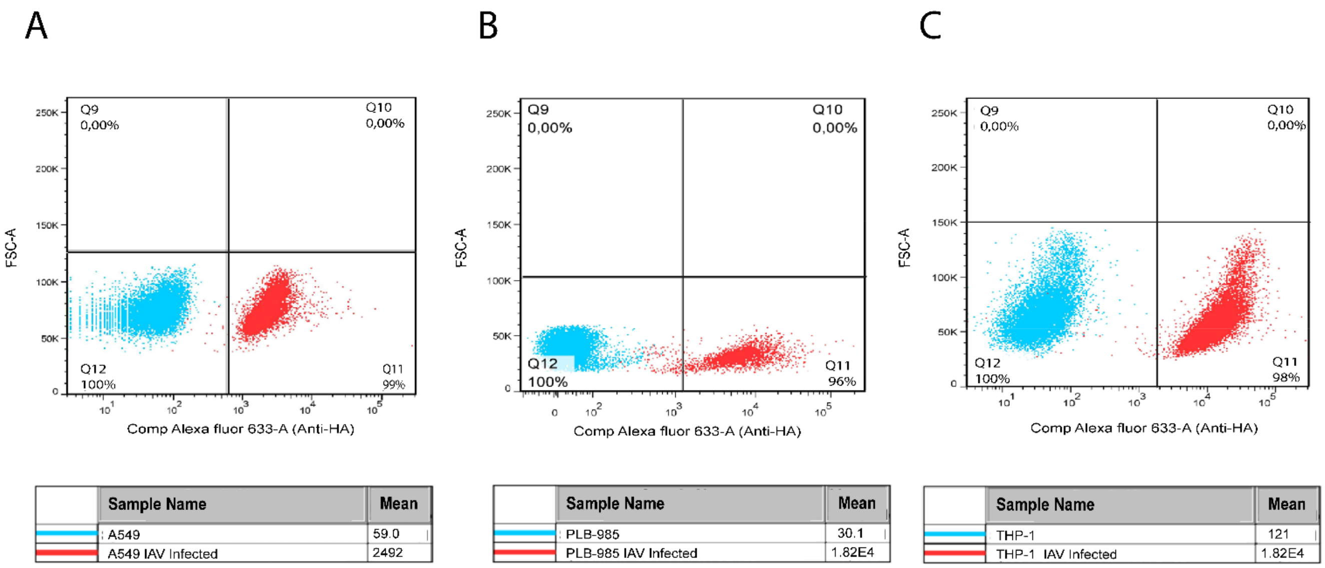

Fig S1. Determination of the percentage of IAV-infected host cells by flow cytometry. A549, THP-1, and PLB-985 cells were infected with IAV (MOI of 10:1) for 24 hours. Subsequently, the cells were washed with PBS and exposed to the anti-HA primary antibody for 1 hour, followed by incubation with the secondary antibody Alexa-Fluor 633 for 30 minutes. The labeled cells were analyzed by flow cytometry, the blue peak corresponds to A549, THP-1, or PLB-985 cells without a signal, while the red peak corresponds to A549 (A), THP-1 (B), or PLB-985 (C) cells labeled with the Alexa Fluor 633 secondary antibody. Data represent at least three independent experiments.

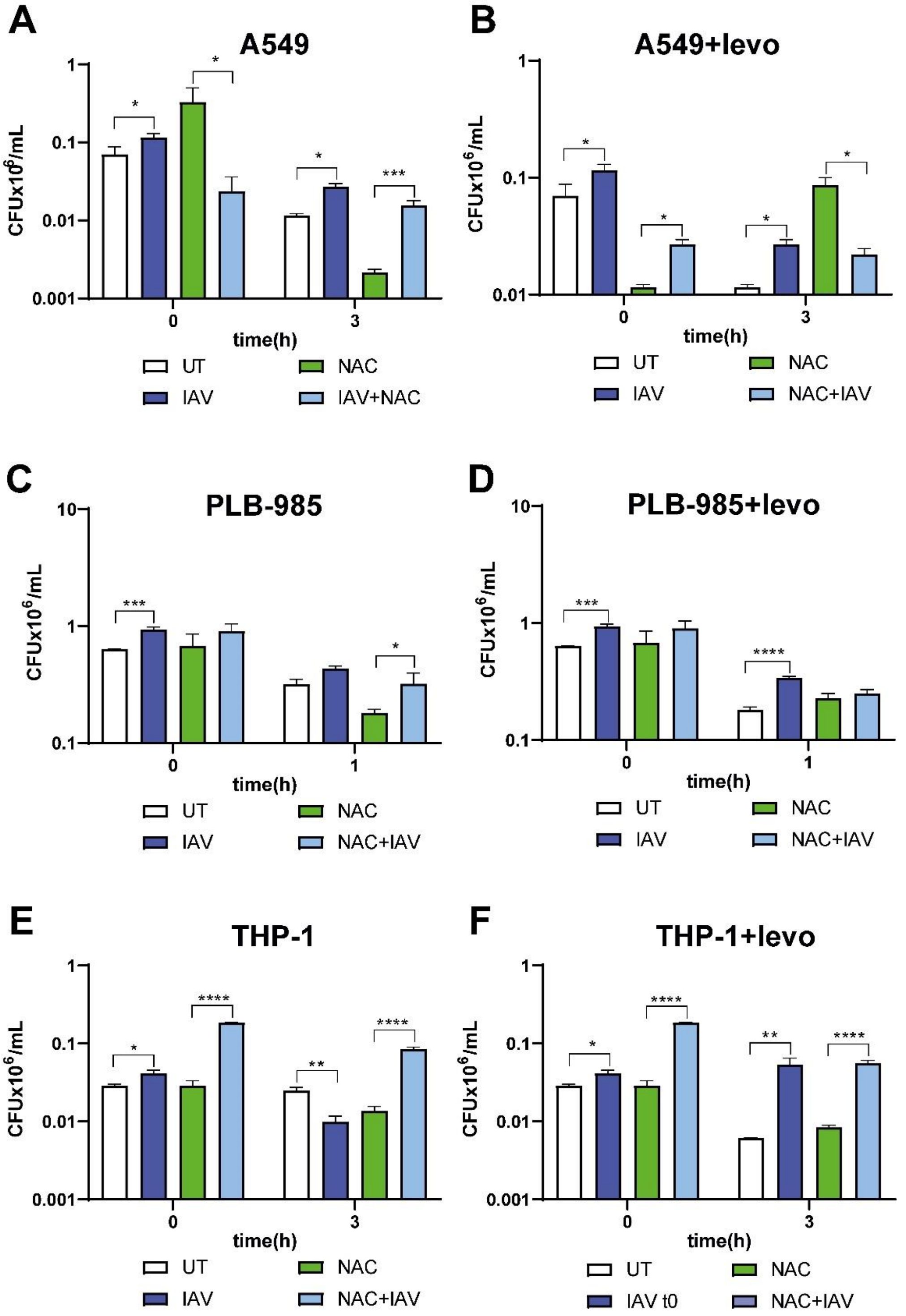

Fig S2. Levofloxacin persistence is increased in IAV-infected host cells. To quantify the *S. pneumoniae* survival, the A549, THP-1 and PLB-985 cells were coinfected with IAV at an MOI of 10 for 24 hours, and then with *S. pneumoniae* for 3h in A549 and THP-1, and 1h in PLB-985 respectively

(See Materials and Methods). The number of CFUs was used to calculate the percentages of survival and FQ persistence during infection of host cells, as shown in Fig 1 and 2. Quantification of colony forming units (CFUs) after infection of A549 (A and B), THP-1 (C and D) and PLB-985 (E and F) cells, with (B, D, and F) and without (A, C, and E) levofloxacin treatment. Data represent at least three independent experiments and informed the FQ persistence percentages in Fig 2A. Error bars indicate standard deviation. Statistical significance (two-tailed test): * $p < 0.05$, ** $p < 0.01$, *** $p < 0.001$, **** $p < 0.0001$.

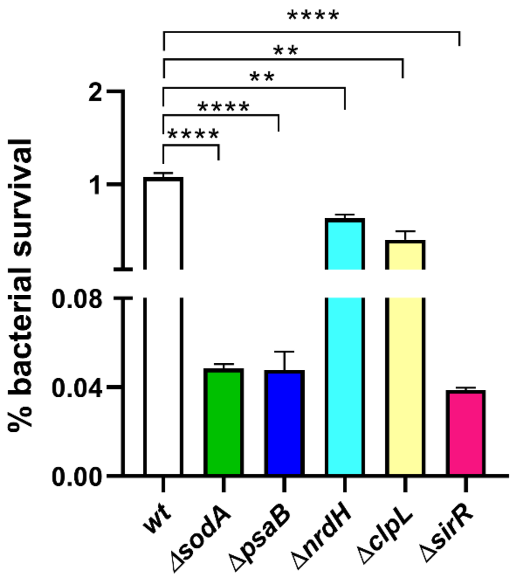

Fig S3. The *ΔsodA, ΔpsaB, ΔnrdH, ΔclpL,* and *ΔsirR* mutants exhibit increased susceptibility to H_2O_2 exposure. Wild-type (*wt*) and mutant bacterial strains were grown to exponential phase ($OD_{600} \approx 0.3$) in BHI broth. Cells were then exposed to 20 mM H_2O_2 for 30 min. Following exposure, bacterial cells were centrifuged (15,000 *g*, 5 min), resuspended in fresh BHI, and serially diluted before plating on blood agar plates. Colony-forming units (CFU) were enumerated after overnight incubation at 37°C. Data represent the mean ± SD of three independent experiments. Statistical significance was determined using a two-tailed Student's t-test (*$p < 0.05$, **$p < 0.01$, ****$p < 0.0001$).

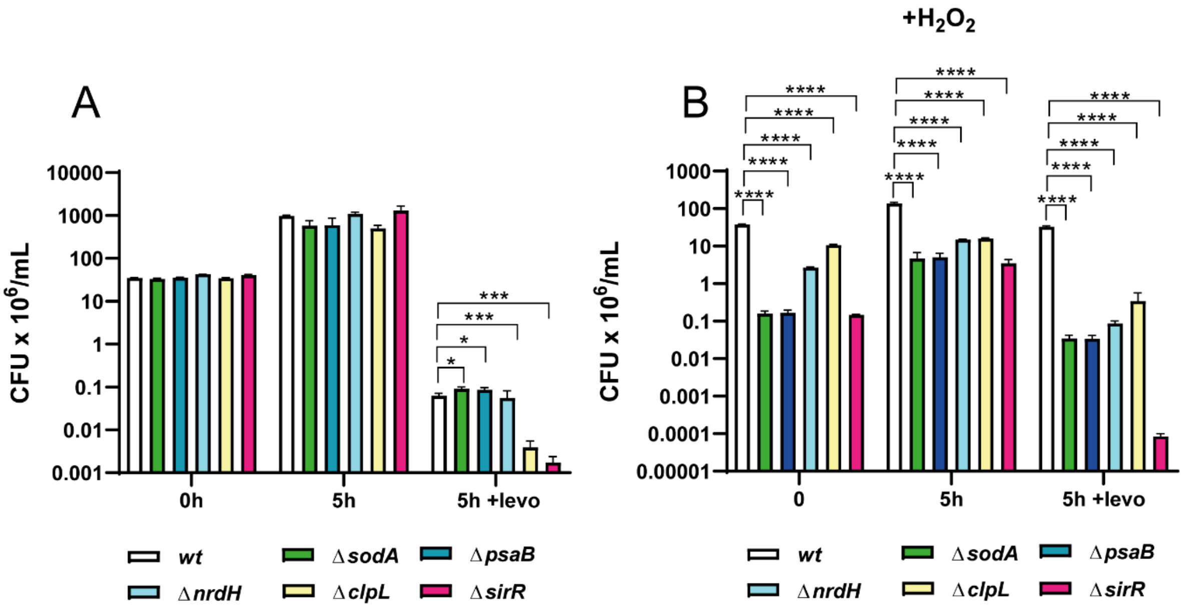

Fig S4. Involvement of *sodA, psaB, nrdH, clpL,* and *sirR* genes in levofloxacin persistence. Wild-type (*wt*) and mutant strains lacking *sodA, psaB, nrdH, clpL,* or *sirR* genes were grown in BHI broth to mid-exponential phase (OD_{600} ≈ 0.3, ~4x10^8 CFU/mL). Bacteria were then exposed to 20 mM H_2O_2 for 30 min. After centrifugation (15,000 x g, 5 min), cells were resuspended in fresh BHI and challenged with levofloxacin (6 μg/mL) for 5 h. Following another centrifugation step, surviving bacteria were quantified by serial dilution, plating on blood agar plates, and incubation at 37°C for 16 h. Colony-forming units (CFU) were counted to assess survival with (A) or without (B) H_2O_2 pre-treatment. These data were used to calculate the percentage of levofloxacin-persisters shown in Fig. 4A. Statistical significance was determined using a t-test: * (p < 0.01), **** (p < 0.0001).

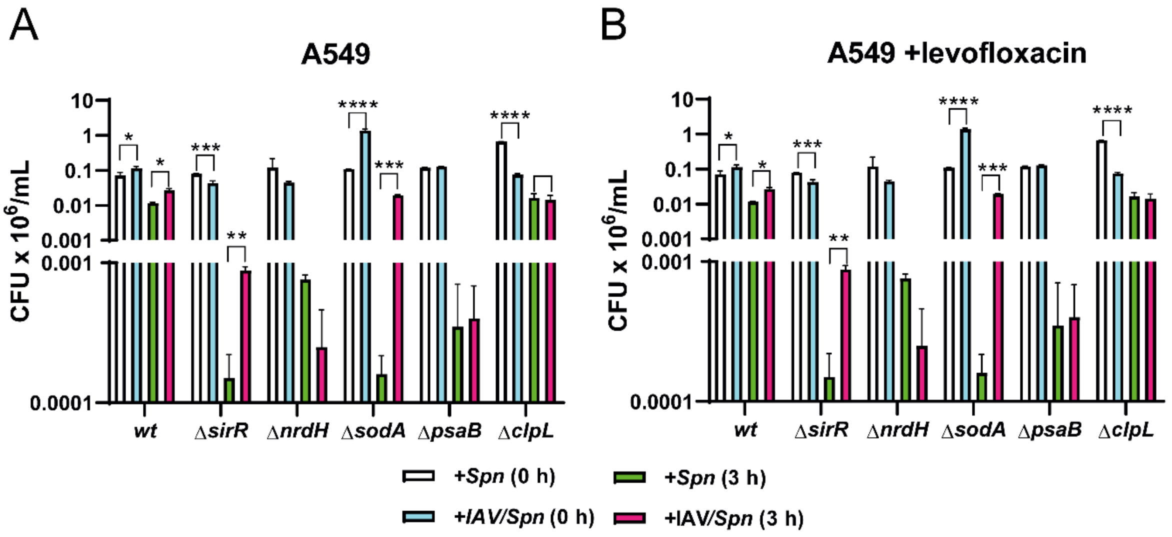

Fig S5. Role of *clpL, sodA, psaB, nrdH,* and *sirR* genes in *S. pneumoniae* levofloxacin persistence during A549 cell infection. Panels show pneumococcal CFU after infecting A549 pneumocytes with or without IAV co-infection, without (A) or with (B) levofloxacin treatment. Data were used to calculate persistence percentages in Fig 4B. Error bars indicate standard deviations ($n \geq 3$). Statistical significance determined by two-tailed test, with * indicating $p < 0.05$, ** indicating $p < 0.01$, *** indicating $p < 0.001$, and **** indicating $p < 0.0001$. Data are representative of at least three independent experiments.

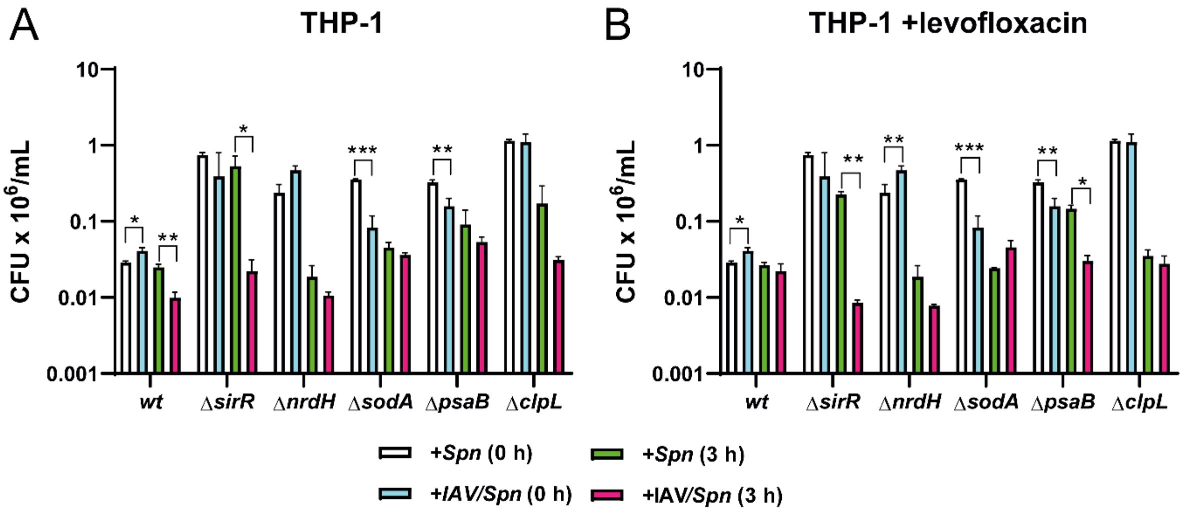

Fig S6. Genes *clpL, sodA, psaB, nrdH,* and *sirR* contribute to levofloxacin persistence in *S. pneumoniae* during THP-1 macrophage infection. Persistence of pneumococcal mutants in THP-1 cells (+/- IAV infection) was assessed as described in Fig. 2 and Materials and Methods. Panels show CFUs after levofloxacin treatment: (A) without treatment, (B) with treatment. These data were used to calculate persistence percentages (Fig 4C). Error bars indicate standard deviation (minimum of three replicates). Significance determined by two-tailed test: *$p < 0.05$, **$p < 0.01$, ***$p < 0.001$, ****$p < 0.0001$. Data represent at least three independent experiments.

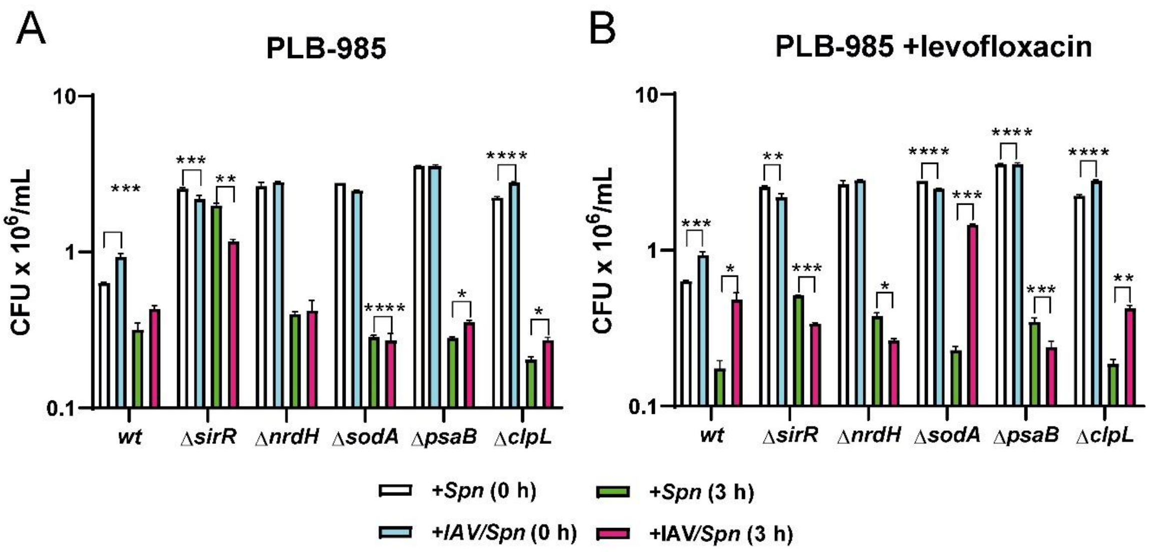

Fig S7. Genes *clpL*, *sodA*, *psaB*, *nrdH*, and *sirR* contribute to levofloxacin persistence in *S. pneumoniae* during PLB-985 neutrophil infection. Quantification of colony forming units (CFUs) after infection of PLB-985 cells without (A) and with (B) levofloxacin treatment, compared to IAV-infected and non-IAV-infected A549 cells (See Fig. 2 and Materials and Methods). Data represent at least three independent experiments and informed the FQ persistence percentages in Fig 4D. Error bars indicate standard deviation. Statistical significance (two-tailed test): * $p < 0.05$, ** $p < 0.01$, *** $p < 0.001$, **** $p < 0.0001$.

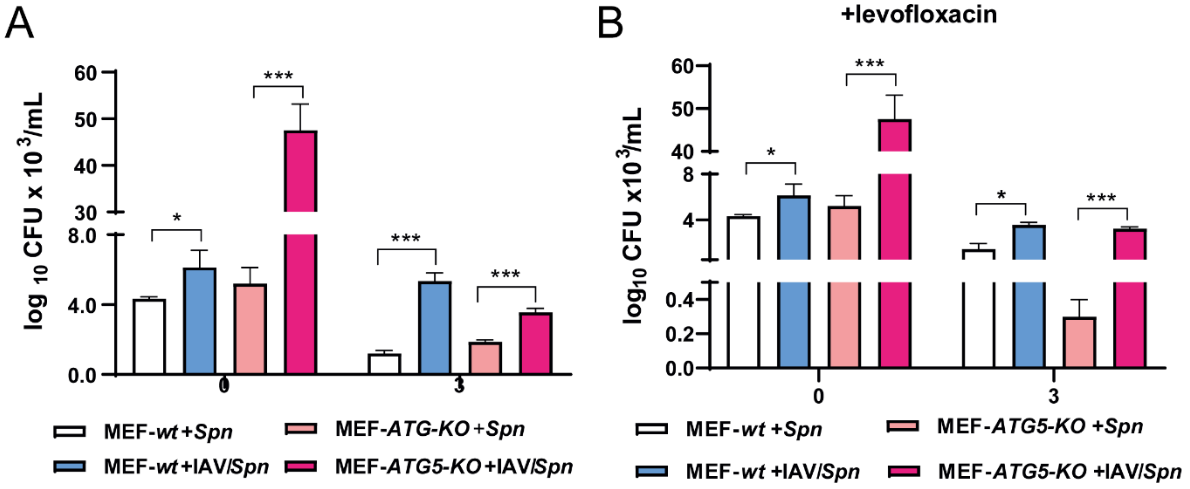

Fig S8. Levofloxacin persistence is increased in autophagy-deficient cells. MEF-*wt* and MEF *ATG5-KO* cells were co-infected with IAV (MOI 10:1, 24 h) followed by *S. pneumoniae* (3 h) as detailed in Materials & Methods. CFU enumeration was used to quantify levofloxacin persistence (see Fig. 4). (A) CFU without levofloxacin. (B) CFU with levofloxacin. Error bars: standard deviation (minimum of three replicates). Significance determined by two-tailed test, **p*<0.05, ****p*<0.001. Data represent at least three independent experiments.

**Table S1.** Bacterial strains, plasmids and primers used in this work		
Name	***Relevant characteristics***	***References***
**Strains**		
R801	Derivative of R6; *hexB-*	1
R806	R801, but *rpsL1*; Sm^R	2
Δ*sodA*	R806, but Δ*sodA:: kan-rpsL+*, obtained from Janus cassette; Km^R, Sm^S	2
Δ*nrdH*	R806, but *ΔnrdH*::kan-rpsL+, obtained from Janus cassette system. The flanking DNA regions were amplified with the FnrdH1, RnrdH1, FnrdH2 and RnrdH2 primers. Km^R, Sm^S	This work
Δ*sirR*	R806, but Δ*sirR:: kan-rpsL⁺*, obtained by Janus cassette system; Km^R, Sm^S	2
Δ*clpL*	R806, but Δ*clpL:: Cm^R*, obtained by CAT cassette; $Cm^{R.}$	2
Δ*psaB*	R806, but Δ*psaB:: kan-rpsL⁺* , obtained from Janus cassette; Km^R, Sm^S	2
**Plasmids**		
pIRES2-EGFP	Vector for expression of a gene and EGFP on one transcript in eukaryotic cells	Novagen
pIRES2-EGFP-M2	Vector for expression of viral protein M2 and EGFP on one transcript in eukaryotic cells	This work
**Primers**		
Fjanus2	TTGGATCCGCTAGCCTCGAGAAGCTTGGAACAAGTT ATTACTTGAAGATGTCAG	*Janus cassette*
Rjanus2	AAGTCGACATCGATAGATCTTCTAGACCCTTTCCTTA TGCTTTTGGAC	*Janus cassette*
FnrdH1	GCCCATTCAGCTTTTTGTTTCG	*nrdH1*
RnrdH1	CATACTCGAGAGGATCCCTCCTCTTCAAAATTTAATA CTATCTTAGTATATCAG T	*nrdH1*
FnrdH2	TCATACTCGAGGGATCCTCTTAGTACATCATCCAGA AGAAACTGC	*nrdH2*
RnrdH2	CCATCAACAGGATTTGCACGG	*nrdH2*

Abbreviations: Km^R, kanamycin resistance; Str^R, streptomycin resistance.

References

1) Lefevre JC, Claverys JP, Sicard AM. Donor deoxyribonucleic acid length and marker effect in pneumococcal transformation. 1979. *J. Bacteriol*. 138:80-6.

2) Reinoso-Vizcaino NM, et al. The pneumococcal two-component system SirRH is linked to enhanced intracellular survival of *Streptococcus pneumoniae* in influenza-infected pulmonary cells. *PLoS Pathog* 16, e1008761 (2020).
